# Wounding Therapies for Prevention of Photocarcinogenesis

**DOI:** 10.3389/fonc.2021.813132

**Published:** 2022-01-07

**Authors:** Timothy C. Frommeyer, Craig A. Rohan, Dan F. Spandau, Michael G. Kemp, Molly A. Wanner, Elizabeth Tanzi, Jeffrey B. Travers

**Affiliations:** ^1^ Department of Pharmacology & Toxicology, Boonshoft School of Medicine at Wright State University, Dayton, OH, United States; ^2^ Department of Dermatology, Boonshoft School of Medicine at Wright State University, Dayton, OH, United States; ^3^ Dayton Veterans Administration Medical Center, Dayton, OH, United States; ^4^ Departments of Dermatology and Biochemistry and Molecular Biology, Indiana University School of Medicine, Indianapolis, IN, United States; ^5^ Richard A. Roudebush Veterans Administration (VA) Medical Center, Indianapolis, IN, United States; ^6^ Department of Dermatology, Massachusetts General Hospital, Boston, MA, United States; ^7^ Capital Laser & Skin Care, Chevy Chase, MD, United States

**Keywords:** non-melanoma skin cancer (NMSC), squamous cell carcinoma, ultraviolet light (UVB), insulin-like growth factor-1 (IGF- I), actinic keratosis (AK), laser resurfacing, chemical peel

## Abstract

The occurrence of non-melanoma skin cancer (NMSC) is closely linked with advanced age and ultraviolet-B (UVB) exposure. More specifically, the development of NMSC is linked to diminished insulin-like growth factor-1 (IGF-1) signaling from senescent dermal fibroblasts in geriatric skin. Consequently, keratinocyte IGF-1 receptor (IGF-1R) remains inactive, resulting in failure to induce appropriate protective responses including DNA repair and cell cycle checkpoint signaling. This allows UVB-induced DNA damage to proliferate unchecked, which increases the likelihood of malignant transformation. NMSC is estimated to occur in 3.3 million individuals annually. The rising incidence results in increased morbidity and significant healthcare costs, which necessitate identification of effective treatment modalities. In this review, we highlight the pathogenesis of NMSC and discuss the potential of novel preventative therapies. In particular, wounding therapies such as dermabrasion, microneedling, chemical peeling, and fractionated laser resurfacing have been shown to restore IGF-1/IGF-1R signaling in geriatric skin and suppress the propagation of UVB-damaged keratinocytes. This wounding response effectively rejuvenates geriatric skin and decreases the incidence of age-associated NMSC.

## Introduction

Skin is the largest and most-exposed organ of the body. It functions as a barrier against environmental pressures, such as ultraviolet (UV) radiation (1). Prolonged and extensive exposure may result in major transformations in skin structure and function, leading to the development of cutaneous pathology (2–4). The American Cancer Society reports that skin cancer is the most commonly diagnosed cancer in the United States (5, 6). Comprised primarily of basal cell carcinoma (BCC) and squamous cell carcinoma (SCC), the exact rates of non-melanoma skin cancer (NMSC) are unknown because most cancer registries do not collect their incidence and mortality data (5). However, it is estimated that 3.3 million are diagnosed each year, resulting in increased healthcare costs for disease management as well as significant morbidity, and rarely, mortality (5, 7). This necessitates exhaustive translational research for innovative treatments and preventative dermo-oncological therapies (8).

The epidemiological link between NMSC, sunlight, and advanced age is well-established, given that at least one in five Americans will develop skin cancer by the age of 70 (9). Moreover, over 80% of individuals with NMSC are older than 60 years of age (10). For some time, the direct causal link between the three observations was unknown, which spurred basic science exploration into photocarcinogenesis. Recent novel findings from our group has implicated the significance of the insulin-like growth factor-1/insulin-like growth factor-1 receptor (IGF-1/IGF-1R) pathway in the development of NMSC (11–13). It has been found that the intensity of UVB exposure directly correlates with the extent of DNA damage. Additionally the regulation between IGF-1/IGF-1R is vital in the protective response and indicative of the tendency for photocarcinogenesis (12, 14–18). Adequate IGF-1 production in dermal fibroblasts is necessary for the appropriate epidermal keratinocyte response to UVB-damaged DNA (11, 12, 19). Geriatric skin exhibits suppressed IGF-1 signaling due to an increased cellular senescence profile of fibroblasts (11, 12, 19). This has severe consequences in the protective response to UVB radiation whereby keratinocytes may have different destinies depending on the extent of DNA damage and state of IGF-1/IGF-1R signaling (11, 12, 19). Activation of keratinocyte IGF-1R induces favorable stress-induced cellular senescence or DNA damage repair such that malignancy prone mutations are arrested without compromising epidermal barrier function (11, 12, 19). However, if IGF-1R is inactive due to diminished IGF-1, keratinocytes may continue to propagate its procarcinogenic DNA damage to its daughter cells (11, 12, 19). This increases the likelihood for development of NMSC (20).

The IGF-1/IGF-1R signaling pathway may be exploited for therapy. In recent years, novel treatments aimed for geriatric patients both predisposed and exposed to NMSC have been developed. One such modality is wounding therapy, which attempts to reverse the geriatric fibroblast senescence phenotype by inducing a “wounding response” (19–23). This effectively serves to rejuvenate geriatric skin to a more youthful phenotypic and constitutive expression. In this review, the therapies of dermabrasion, microneedling, chemical peeling, and fractionated laser resurfacing will be discussed in the context of prevention of photocarcinogenesis.

## Background of Non-Melanoma Skin Cancer

It is estimated that more people are diagnosed with skin cancer than all other types of cancer combined (5). In addition, between 1976-1984 and 2000-2010, epidemiologic research reveals an increasing overall incidence of 145% and 263% for BCC and SCC, respectively (24). Consequently, skin cancer is a major public health concern as the associative healthcare costs are extensive and rising (25). The increasing prevalence and economic burden underscore the need to understand the risks factors and etiology of NMSC.

As with other neoplastic pathologies, NMSC is of multifactorial origin. The exact mechanism of development is not well defined, but is likely due to the interplay of environmental, genetic, and phenotypical factors (26). NMSC is characterized by multiple risk factors that are both endogenous and exogenous in nature. The endogenous risk factors include patients with light-colored skin and blue eyes, Fitzpatrick Skin Phototypes I-III, presence of dysplastic nevi, evidence of family history, and genetic conditions such as oculocutaneous albinism and xeroderma pigmentosum (27). Furthermore, exogenous factors include infection by human papilloma viruses (HPV), sun protection behavior, history of sunburns, and magnitude of UV exposure (27). Lastly, individuals who are of advanced age, immunosuppressed, and demonstrate a chronic inflammatory state are also at risk for development of NMSC. Among the aforementioned risk factors, environmental exposure to UV light as well as advanced age are the most commonly acquired and will be the focus of this review.

### Sunlight and Advanced Age

There is considerable evidence that substantiates sunlight and advanced aging as a likely cause of NMSC (16, 28–30). These studies include increased incidence in sunnier cities and those exhibiting a lifestyle of prolonged sunlight exposure, lower prevalence in darker skin phototypes, and a majority of cases occurring over sun-exposed skin and in those older than 60 (10, 31–33). In addition, skin cancer exhibits a strong correlation with age, such that nearly 80% of cases occurs in patients over the age of 60 (10). For a while, the epidemiological link between NMSC, sunlight exposure, and advanced age lacked direct causality, prompting basic science research to discover viable connections. UV exposure is known to induce the formation of reactive oxygen species (ROS) and cyclobutane pyrimidine dimers, often resulting in DNA mutations (34). Epidemiological evidence suggests that excessive sun exposure in the first two decades of life can lead to UVB-induced mutations in keratinocytes (35–38). It was thought that this DNA damage persists in the epidermis, eventually obtaining a growth advantage supporting skin carcinogenesis over many decades. However, recent literature suggests that almost 80% of lifetime exposure to sunlight occurs after the age of 18 (10). In addition, it is well-known that sunscreen use is protective against photocarcinogenesis, which suggests that the acquisition of skin cancer is an ongoing process (39–41). Since aging is also associated with a diminished ability to repair DNA, it is reasonable to assume that this component of aging contributes to skin carcinogenesis (11, 42, 43). Overall, studies ultimately support the association between UVB injury, advanced age, and NMSC.

### Dermal Microenvironment

Human skin consists of an outer epidermal layer and inner dermal layer connected by a basement membrane as well as underlying subcutaneous fat (27, 44). Keratinocytes are the predominant cell in the epidermis, which is made up of four sub-layers (27, 44, 45). These cells proliferate in the basal layer while attached to the basement membrane. Once detached, keratinocytes stop dividing and undergo a final differentiation known as cornification (44, 45). Each epidermal sub-layer represents a different stage of keratinocyte maturity, whereby they function to strengthen the cytoskeleton as well as establish an epidermal protective barrier (27, 44, 45). The underlying dermis provides support and nutrients for the epidermis (27, 44). It is characterized by a lower cellular density and extensive extracellular matrix. In addition, the dermis is divided into two layers: the more superficial papillary layer and the deeper reticular layer (44). The papillary layer is densely populated by fibroblasts, which are the dominant dermal cell (44). The fibroblast cells of the adult dermis are specialized, post-mitotic, and non-proliferative, while epidermal keratinocytes are highly active and continuously dividing to renew the outer skin barrier (44–46). Since dermal fibroblasts are a long-lived cell population, they experience unceasing damage accumulation and adaptation processes that are associated with aging (44–46). Conversely, the epidermis experiences the direct effects of environmental exposure, which adds to senescent processes (44–46). Thus, cell aging and senescence of epidermal keratinocytes and dermal fibroblasts are largely implicated in skin aging.

Multiple studies have shown that the pathogenesis of photoaging is also associated with supportive tissue stroma, effectors of the immune system, diminished melanogenesis, inappropriate fibroblast deposition, and cytokines and growth factors (47, 48). Tumor growth and progression is dependent on its permissive microenvironment (49). One factor lending to the development of photocarcinogenesis is the senescence-associated secretory phenotype (SASP) (49–51). Senescent cells demonstrate not only an arrest of cell proliferation, but also high metabolic activity marked by widespread changes in protein expression and secretion (49, 50). This can lead to increased transcription of cytokines such as IL-1, IL-6, IL-8, MMP-1, and MMP-3, resulting in chronic low-level inflammation and disruption of normal physiologic processes (19, 49–51). Thus, the molecular profile of the senescent dermal microenvironment plays a major role in skin carcinogenesis. Importantly, the IGF-1/IGF-1R signal transduction pathway has been recently established as a major mechanism in the development of NMSC in the elderly (11, 12, 18). These findings highlight the complexity of NMSC and suggest its development is a gradual and sun-induced process (39–41).

## Pathogenesis of Non-Melanoma Skin Cancer

Aging and excessive UV exposure are two of the main drivers in the development of NMSC. For a while, the exact mechanisms underpinning how a lifetime of excessive sunlight exposure and advanced age lead to the development of skin cancer were not well understood. However, data from our laboratory suggested a new paradigm for the role of aging in photocarcinogenesis involving the IGF-1/IGF-1R signaling pathway, which regulates the cellular response of keratinocytes to UVB exposure (52). These studies propose a mechanism where geriatric skin deficient in IGF-1 expression is unable to activate the IGF-1R in keratinocytes, resulting in an aberrant response to UVB irradiation (11, 12). This leads to epidermal keratinocytes passing its UVB-induced DNA damage onto daughter cells, likening the formation of NMSC (11, 12, 18, 52).

### UVB-Exposure and the Epidermis

Sunlight is composed of multiple types of light including infrared, visible, and ultraviolet (UV) (14). UV light is further classified into UV-A, UV-B, and UV-C. Wavelengths of light within the UV-A range are known to penetrate the atmosphere; although, its impact is indirect and facilitated by free radical formation (38, 53, 54). Conversely, most light in the UV-C range is absorbed in the atmosphere, limiting its dissemination to the earth’s surface (14). UV-B comprises only 0.3% of the total light that reaches the surface of the earth; however, its wavelengths penetrate the outermost layer of the skin (15). Though limited to the epidermis, UV-B can directly damage keratinocyte DNA through induction of pyrimidine dimers and other DNA photoproducts that are potentially mutagenic (14, 53, 55–57). These mutagenic changes have the potential to be propagated to subsequent cellular populations, raising the possibility of pro-carcinogenic changes (58–60). The human body has repair mechanisms that respond to UV-B radiation; however, the extent of the epidermal response is largely dependent on the dose and duration of UV-B (53, 61, 62). Short-lived and low dose exposures spur DNA repair by temporarily halting the cell cycle of keratinocytes (11, 56). High doses of UVB cause extensive DNA damage, which results in apoptosis of keratinocytes (11, 56). In contrast, prolonged and intermediate doses of UV-B radiation results in enhanced DNA damage that may escape DNA repair and lead to pro-carcinogenic cellular proliferation (11, 56).

Humans possess a system for removing UV photoproducts from mutated DNA known as nucleotide excision repair (NER) (63). This repair system functions through removal of damaged DNA bases and repair of the gaps by the actions of DNA polymerase and ligase (63). Supplementary to NER, cells possess additional systems such as DNA damage checkpoints that detect the presence of UV photoproducts and control DNA replication and cell cycle progression (63). In particular, the ATR-CHK1 signaling network acts to transiently suppress DNA synthesis in UVB-damaged cells through the suppression of DNA synthesis and cell cycle progression by the G1-S DNA damage checkpoint (63). However, keratinocytes can sometimes escape the appropriate DNA repair. For those basal keratinocytes that experience intermediate doses of UVB-damage, several outcomes can occur: (1) apoptosis of the damaged cell, (2) pro-carcinogenic cellular proliferation, or (3) keratinocyte senescence as a tumor evasion mechanism (11, 12, 18, 19). The first outcome has the benefit of removing the potential pro-carcinogenic agent, though at the expense of damaging the epidermal barrier function. It should be noted that all three outcomes can be modulated by the activation status of the IGF-1/IGF-1R signaling pathway. The keratinocyte response serves as a protective mechanism.

### Aging, Fibroblast Senescence, and Photocarcinogenesis

**Figure 1:**
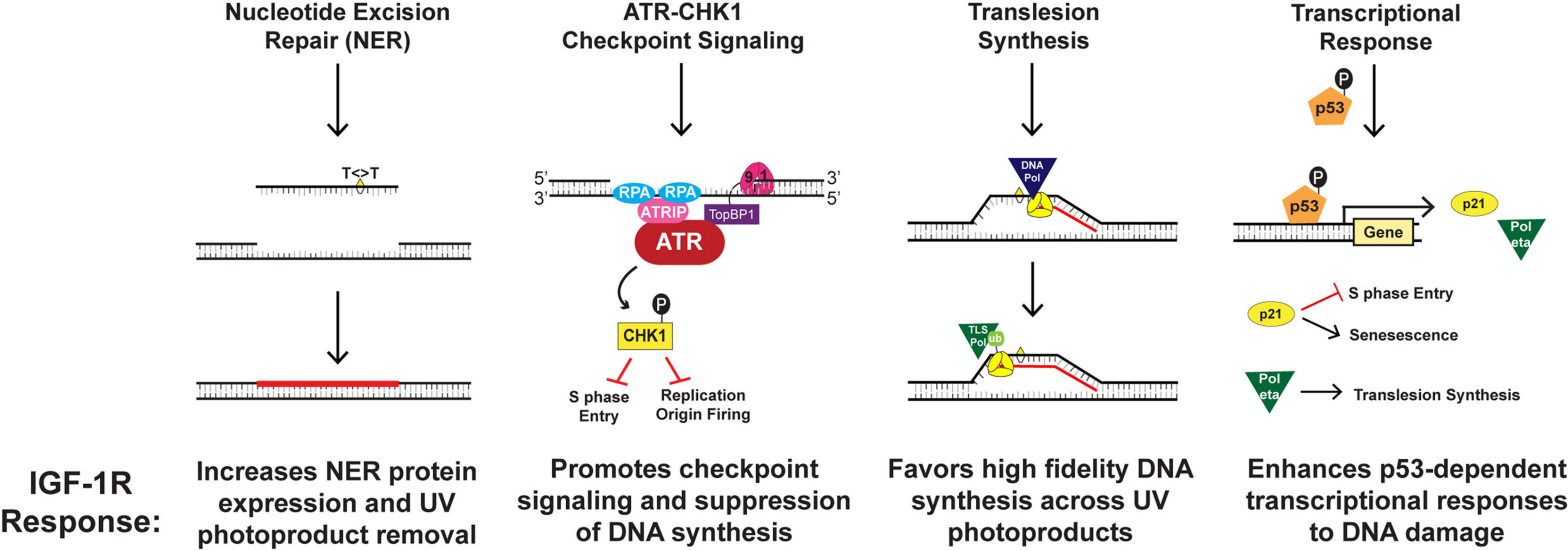
Mechanisms by which IGF-1R activation results in keratinocyte protection from UVB-irradiation.

The IGF-1/IGF-IR mechanism is compartmentalized within dermal-epidermal interactions, wherein dermal fibroblasts regulate basal keratinocyte differentiation (11, 12, 19). IGF-1R is a tyrosine kinase receptor expressed on epidermal keratinocytes that is activated by IGF-1, which is produced and secreted by papillary dermal fibroblasts (64). This signaling pathway is maintained throughout one’s lifetime to allow the maintenance and growth of healthy skin (11, 12, 19). Among normal cellular upkeep, IGF-1R activation by IGF-1 plays an integral role in DNA replication and repair, checkpoint control, and induction of keratinocyte senescence (11, 63, 65, 66). Yet, stromal changes in aging skin can impair these processes. Aging causes a number of changes to skin morphology and physiology, including a decrease in epidermal thickness and cell turnover (63). More importantly, the aging process considerably alters IGF-1 synthesis and secretion from dermal fibroblasts (11, 12, 19). Consequently, the paucity of IGF-1 ligand in aged skin results in diminished activation of IGF-1R on keratinocytes (11, 12, 19). Outlined in **Figure 1**, four important components of the cellular DNA damage response are debilitated by the aging process in keratinocytes, including UV photoproduct removal by NER, checkpoint signaling through ATR-CHK1, the replication of damaged DNA by translesion synthesis (TLS), and the induction of gene products regulated by the tumor suppressor protein p53 (**Figure 1**) (18, 52, 65–68). Together, this altered DNA damage response results in keratinocytes that fail to undergo senescence and instead continue to proliferate (11, 12, 65, 66).

The ATR-CHK1 kinase signaling pathway acts to transiently suppress DNA synthesis and cell cycle progression when UVB-irradiation damages cellular DNA (63, 65). This signaling cascade works through various mechanisms including halting the progression from G1 to S phase of the cell cycle, decelerating the rate of ongoing replication fork progression, and hindering the initiation of DNA replication at new origins (63, 65). Our group studied that IGF-1R activation affects ATR-CHK1 signaling through introduction of small-molecule inhibitors or IGF-1 withdrawal (65). We found that disturbing the IGF-1/IGF-1R signaling pathway resulted in redaction of the ATR-CHK1 activation cascade and consequent failure to inhibit chromosomal DNA synthesis in UVB-damaged keratinocytes (65). This indicates that geriatric skin carcinogenesis in lieu of deficient IGF-1 signaling may be caused by defects in cellular responses to UVB-damaged DNA such as suppression of DNA synthesis and cell-cycle progression (65). Similarly, our group studied whether DNA damage tolerance systems including TLS are altered in geriatric individuals (66). The TLS pathway is known to recruit specialized DNA polymerases to damaged DNA, which can introduce nucleotides opposite to damaged template DNA in an error-prone manner (63, 65). Monoubiquitination of the replicative DNA polymerase clamp protein PCNA (proliferating cell nuclear antigen) was used as a biomarker of TLS pathway activation (66). UVB-exposure on geriatric (age >65) skin resulted in a higher level of PCNA monoubiquitination than that found in younger skin (66). Notably, both pharmacological inhibition of IGF-1R as well as IGF-1 deprivation potentiated UVB-induced PCNA monoubiquitination (66). Though the TLS polymerase pol eta can accurately replicate UV photoproducts, we found that its induction by UVB exposure is partially abrogated by the loss of IGF-1 signaling in keratinocytes and human skin explants (68). This suggests that altered IGF-1/IGF-1R signaling in aged skin may predispose keratinocytes to undergo a more mutagenic form of DNA synthesis after UVB-exposure (66).

Appropriate IGF-1/IGF-1R signaling is critical in the dermal response to sun-damaged skin (8). As mentioned before, intermediate doses of UVB can result in keratinocytes escaping effective DNA repair (11, 56). Keratinocytes in this scenario can either undergo apoptosis, cell proliferation with UVB-damaged DNA, or survive through cellular senescence (11). The last option yields the best outcome such that the epidermal skin barrier maintains its function and potentially mutagenic cellular proliferation is halted. However, this signaling pathway loses its viability in geriatric skin, so that the reduced IGF-1 expression results in an inappropriate response to UVB-damage (11, 12). Since keratinocyte IGF-1R activation is dependent on its ligand, the paucity of IGF-1 in aged dermis results in pro-carcinogenic replication of UVB-damaged DNA (11, 12). In younger skin, IGF-1 synthesis and secretion is sufficient to uphold normal physiological activity of IGF-1R (11, 12). Thus, if UVB-damaged DNA of young keratinocytes is not fully repaired, then keratinocyte senescence stands as a tumor evasion mechanism (8, 11, 12). Unfortunately, when geriatric skin experiences UVB-irradiation, a portion of the epidermal keratinocytes respond inappropriately by allowing mutagenic DNA to replicate and potentially initiate neoplastic cells (8, 19). Consistent with this notion, recent studies by our group xenografted human skin onto immunodeficient mice, and the human skin grafts underwent a chronic UVB carcinogenesis protocol with/without treatment with a topical IGF-1R inhibitor. A 20 week UVB treatment of the skin treated with IGF-1R inhibitor, which would mimic geriatric skin, resulted in formation of actinic neoplastic (AKs/early SCC) lesions, findings not seen in vehicle irradiated skin (20). The data from these studies suggests that defective IGF-1/IGF-1R signaling due to senescent dermal fibroblasts is an important cause of geriatric NMSC.

## Current Treatment Modalities

Treatment options for NMSC depends on risk stratification of the tumor and its characteristics, availability of services, and patient preference and suitability (26, 69). There is a lack of high-quality and evidence-based studies with a 5-year follow-up for NMSC management (26). Additionally, the risk of recurrence after treatment is high, though these cancers seldom metastasize (26, 70). Thus, systemic treatments are not regularly of importance (70). Surgery has traditionally been the “gold standard” treatment due to its excellent cure rates and desirable cosmetic results (70, 71). Specifically, Mohs micrographic surgery (MMS) is the benchmark for high-risk lesions and locations (69). There are other non-surgical treatment modalities such as physical destruction (cryotherapy, radiotherapy, and curettage and cautery), chemical destruction (photodynamic therapy), local therapies (topical 5-fluorouracil, imiquimod, ingenol mebutate, and diclofenac), and novel hedgehog pathway inhibitors (HPI) (vismodegib and sonidegib) (71, 72). The recent development of HPI may antecede a shift towards medical management of NMSC (71).

The annual healthcare cost of treating NMSC in the United States is estimated at nearly $5 billion, which has encouraged exploration to identify and develop effective therapies (25). Most of the current therapies are appropriate only after tumors are clinically manifested and fail to attenuate the rising economic burden (8). However, therapies focused on prevention are successful in preventing pre-malignant transformation. Such is the case in treatments for actinic keratoses (AK), which are pre-cancerous lesions that may evolve into SCC (73). Successful therapies include cryotherapy, photodynamic therapy, 5-fluorouracil with or without topical calcipotriol, topical imiquimod, electrosurgery, and curettage (74–76). These modalities have revealed that early treatment of AK prevents the progression to SCC (74–76). More recently, the combination therapy of calcipotriol and 5-fluorouracil was effective in preventing SCC development within 3 years after treatment (76). Despite the success of AK treatment modalities, these therapies only focus on averting transformation of already established pre-cancerous lesions. Early treatment of AK is essential in preventing progression to SCC (77). As a result, untreated and histologically unaffected skin remains vulnerable to malignant transformation in at-risk individuals (8). This has oriented novel treatments towards skin rejuvenation and dermal IGF-1 restoration strategies, such as wounding therapy (8).

## Wounding Therapies for Rejuvenation

The critical observations implicating deficient IGF-1/IGF-1R signaling in NMSC have led to the development of new potential targeted therapies. These treatments act by wounding the skin, which effectively restores IGF-1/IGF-1R signaling by reversing geriatric fibroblast senescence (21–23, 52, 77). Thus, wounding therapy has the potential to achieve an efficacious and long-term chemo-preventative effect, which allays the rising healthcare burden and morbidity associated with NMSC (21–23, 52, 77). In addition, these skin rejuvenation modalities are able to attain both cosmetic and cancer prophylactic effects (8, 78). Our group has identified four viable wounding therapies: dermabrasion, fractionated laser resurfacing (FLR), microneedling, and chemical peeling (19, 21–23, 63). However, the exact mechanism as to how wounding strategies are able to rejuvenate geriatric skin and prevent malignant transformation is unknown (77). Further translational research is needed to explicate the direct pathways.

### Dermabrasion

Dermabrasion is a resurfacing technique that has been in use for over 100 years to treat a variety of dermatological conditions, such as scar revision, facial skin resurfacing, wound healing, and correction of pigmentary abnormalities (79–81). The technique is performed using a portable hand-held dermabrader with either wire brushes, diamond fraises, or serrated wheels attached for precise treatment (80). Additionally, sterilized sandpaper or sterile electrocautery scratch pads have been used to manually abrade the skin (79). Dermabrasion intentionally and selectively damages skin to promote reepithelialization and the production of dermal collagen fibrils (8, 19, 79, 81).

Accordingly, our group assessed if dermabrasion was a viable modality to upregulate IGF-1 expression and restore the appropriate UVB response (19). We recruited geriatric volunteers (age > 65 years) and dermabraded discrete areas of their skin, with complete removal of all epidermis and superficial dermis (2, 19). Three months later, the treated loci were irradiated with UVB; and a punch biopsy was performed on the irradiated site as well as adjacent unirradiated skin for histological and biochemical analysis (19). Our group discovered that dermabrasion produced scarce senescent fibroblasts and fully restored levels of dermal IGF-1 mRNA in geriatric skin (19). Moreover, we found histological features characteristic of younger skin including elliptical fibroblast-replicating nuclei, denser distribution of fibroblasts, restoration of dermal collagen, recovery of the undulating dermal-epidermal basal membrane, and increased number of proliferative keratinocytes (19). Significantly, IGF-1 levels were restored to a profile comparable to skin found in young adults (age < 30) and there was no evidence of UVB-damaged basal keratinocytes (19). As a result, our group was first to demonstrate that dermabrasion restores a more youthful phenotype and induces a reversible molecular signature that can suppress the typical geriatric pro-carcinogenic UVB response (19). Although a promising therapy, the outcomes of dermabrasion are largely dependent on the appropriate technique and skill of the operator; and may produce potentially unfavorable cosmetic outcomes (79–82). For this reason, our group investigated whether other effective but less aggressive wounding modalities, such as FLR and microneedling, could restore an appropriate geriatric UVB response.

### Fractionated Laser Resurfacing

FLR has been used over the past two decades for photoaging, acne scarring, and dyschromia (83, 84). The fractionation allows for deeper tissue penetration, and results in tissue remodeling and collagen production (83). FLR delivers infrared light to tissue where it is absorbed by water in the dermis (8). This causes skin heating at non ablative wavelengths such as 1470 nm, 1540 nm, 1550 nm, and 1927 nm and vaporization of tissue layers at ablative wavelengths including 2940 nm and 10,600 nm, which induces skin wounds (8, 85). By thermally altering a distinct area of the skin, the adjacent and untouched skin is able to quickly repopulate the ablated columns of tissue through increased fibroblast and epidermal stem cell activity (85). In addition to requiring less technical skill than dermabrasion, FLR results in more favorable cosmetic outcomes and rapid wound healing (79, 80, 84).

Our group explored whether a FLR-induced wounding response would correct the inappropriate UVB response in geriatric skin (21). Geriatric volunteers (age > 65) received FLR on either sun-protected skin or chronically sun-exposed skin (21). After three months, the FLR-treated skin was irradiated with UVB, and biopsies were taken from irradiated and unirradiated adjacent skin (21). Independent of skin sun exposure, we found increases in collagen expression, amplified numbers of fibroblasts, upregulation of dermal IGF-1 expression, and restoration of the normal UVB response (21). In addition, there was a reduction of photodamaged keratinocytes in chronically sun-exposed skin (21). These results were similar to that of dermabrasion, but with much more desirable cosmetic effects and procedural ease (79, 80). This suggests that FLR could also be used to help protect against future actinic neoplasia (77). Therefore, our group explored the use of single ablative FLR as a modality to treat AK (23). Subjects (age > 60) with at least five AKs on the forearm or wrist received FLR treatment (23). At three and six months, the treated sites were photographed and had the AK lesions counted and mapped in a blinded fashion (23). When compared to pre-treatment, we found that the numbers of AKs on treated locations were significantly lower at both three months and six months (23). Additionally, the average total numbers of AKs on the untreated arm at six months increased by 167%, while the average percentage decrease of AKs on the treated arm was 60% (23).

The results demonstrate the utility of a single FLR treatment as a field therapy to treat precancerous AKs on sun-exposed skin, given its upregulation of dermal IGF-1 and removal of senescent fibroblasts (21, 23). However, these studies only examined responses within a short time frame post-wounding. In addition, the safety, efficacy, and durability of single ablative FLR in high-risk geriatric patients is unknown. Thus, our group assessed the long-term effects of FLR on geriatric skin through a randomized prospective clinical trial. We recruited 48 patients (age > 60) who had at least five AKs that were 3 mm or smaller. Patients underwent a single treatment of FLR on the upper extremity of aged skin. They were examined at three month post-wounding and every six months thereafter for a current 36-month follow-up period (20). To determine the effectiveness of FLR in reducing the occurrence of AKs, the ratio of the number of AKs on FLR-treated arms to untreated arms was tracked. At three months post-FLR treatment, the ratio of AKs on treated versus untreated arms was reduced four-fold. This ratio was maintained throughout the current 36-month period, demonstrating a lack of significant difference in the ratios at 3, 6, 12, 18, 24, 30, or 36 months. Moreover, additional analyses were conducted to model the initiation of new AK lesions. We found that untreated arms continue to accumulate increasing numbers of AKs, while treated arms demonstrated a reduction in the occurrence of new AK lesions with time. In fact, the number of AKs on untreated arms accumulated at a much faster rate than that found on treated arms. The results not only indicate that FLR is an effective treatment for existing AKs, but also prevent the development of new AK lesions. Importantly, the numbers of NMSC on the untreated arms of this population (26 NMSC) was much greater than the arm that underwent FLR (2 NMSC). Moreover, with efficacy lasting for at least two years following treatment, we validate that FLR is a durable treatment for rectifying the inappropriate UVB response in elderly skin. This data suggests that a single treatment of FLR can provide lasting prevention of NMSC in high-risk geriatric patients (20).

### Microneedling

Microneedling is a reasonably new treatment modality within the field of dermatology, largely being used for aesthetic purposes (86, 87). It has a broad range of uses including acne and surgical scarring, melasma, rhytides, dyschromia, transdermal drug delivery, and skin rejuvenation (86, 88). Microneedles are reported to be both effective and versatile devices due to its relatively painless penetration, affordability, and ability to deliver transdermal medicinal applications (88). The basic theory behind its mechanism of action is percutaneous collagen induction (78, 87). Histological studies exhibit increased collagen and elastin after use of a microneedling device, which introduces small zones of dermal injury and subsequent wound healing processes (87). Previous studies by our group have exhibited photorejuvenation through wounding therapies such as dermabrasion and FLR (19, 21, 23). Given its rising popularity for skin rejuvenation, our group tested if controlled microneedling could achieve a similar protective wounding response as exhibited by upregulation of dermal IGF-1 levels (22).

Nine geriatric volunteers (age > 65) with Fitzpatrick Types I and II underwent wounding on the upper buttocks using a commercially available microneedling device (22). After 90 days, a localized area of either microneedle-treated skin or untreated skin was irradiated with a dose of UVB (22). Photographs and punch biopsies of the skin were acquired twenty-four hours post-UVB irradiation (22). At three months after microneedling application, mRNA levels of both collagen 1 and IGF-1 were increased in previously wounded skin (22). These results were similar to that found after application of dermabrasion (19, 22). When comparing UVB irradiated wounded skin versus UVB irradiated normal control skin, we found a statically significant decrease in the numbers of Ki67+/TD+ basal keratinocytes in wounded skin (22). This paralleled the responses exhibited following dermabrasion and FLR wounding of elderly skin (12, 19, 21). Furthermore, this response to UVB irradiation was similar to the “normal” responses documented in young (age < 30) skin (12, 19, 21, 22). This study indicates that wounding of geriatric skin by use of a microneedling device results in increased dermal collagen 1 and IGF-1 levels as well as normalizes the protective response to UVB irradiation. These findings are promising, but are limited to a short time-frame and small sample size.

### Chemical Peels

Chemical Peeling, also known as chemexfoliation, has been used for centuries to attenuate photoaging and holds promise as another skin resurfacing option (89, 90). This modality utilizes a chemical application to the skin that causes controlled wounding of the epidermis and dermis, resulting in skin regeneration (89). The extent of wounding depends on the depth of skin penetration, thus it is characterized as superficial, medium-depth, or deep chemical peels (91). Superficial peels produce wounding limited only to the epidermis, while medium-depth peels penetrate into the papillary dermis. Moreover, deep peels generate injury into the reticular dermis (89, 91). These peels are indicated for a number of skin conditions, including acne, melasma, actinic keratosis, lentigines, photodamaging, and scarring. Additionally, a number of chemicals are used for peeling, such as tretinoin, salicylic acid, trichloroacetic acid (TCA), Jessner’s solution (JS), glycolic acid (GA), pyruvic acid and phenol (89, 91). The use of superficial and medium-depth chemical peels has increased in recent years due to its relative procedural ease, minimal side effects, and cost efficiency (92). Thus, an investigation into its potential as a wounding therapy for prevention of photocarcinogenesis is warranted.

There are a number of human studies that suggest chemical resurfacing may be a viable skin cancer prophylaxis (93–98). Using a human keratinocyte cell line, Ahn et al. demonstrated the ability of GA to inhibit UV-induced cytotoxicity and apoptosis, which suggests GA may exert a repressive effect on skin cancer development (93). Kaminaka et al. investigated the efficacy of phenol peels in patients with AK and Bowen disease. They found that 100% pure phenol resulted in a 84.8% complete response after one to eight treatment sessions, with only 4.3% recurrence over a period of one year (94). Lawrence et al. found that a medium-depth peel using JS and 35% TCA reduced the number of visible AK by 75% (95). Similarly, Hantash et al. found that 30% TCA resulted in a 89% clearance of AKs when measured at three months post therapy. Furthermore, this application demonstrated a reduced incidence of NMSC compared to the control as well as a longer timespan until the development of new skin cancer (96). Lastly, two recent studies compared TCA peeling (35% and 50%) and photodynamic therapy with topical 5-aminolevulinic acid (PDT-ALA) for the treatment of AK (97, 98). Although TCA was less painful and less expensive, both studies found that PDT-ALA performed better than TCA. Yet, Di Nuzzo et al. found that 50% TCA had an AK clearance rate of 66.1% at three and six months (97). In addition, Holzer et al. demonstrated that 35% TCA had a 78.6% and 48.8% AK clearance at three and twelve months, respectively (98). This suggests TCA peels have clinical utility in treating AKs. Our group is currently exploring TCA peels in this regard, and our preliminary studies indicate that a 10% TCA peel on geriatric skin upregulates IGF-1 mRNA levels approximately two-fold at 90 days (*unpublished data*). As presented, chemical peeling holds promise as a therapeutic and preventative modality for skin carcinogenesis. More research is needed with this area to elucidate the precise mechanize of skin rejuvenation. Chemical peeling offers a safe, cost-effective and flexible alternative wounding therapy for the prevention of photocarcinogenesis.

## Conclusion

**Figure 2:**
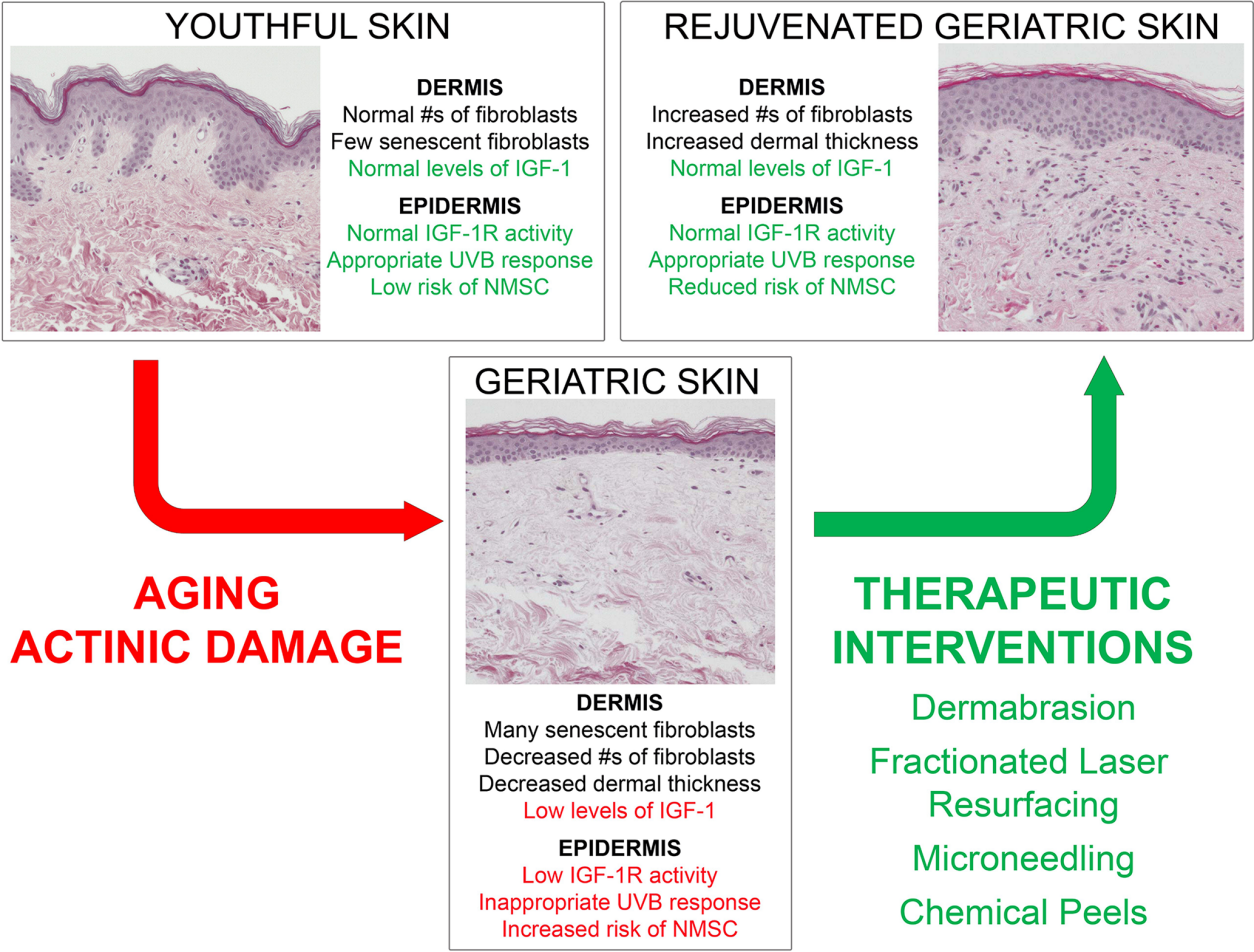
Model demonstrating young versus aged skin and the impact of wounding therapies on the response of geriatric skin to UVB-irradiation.

The IGF-1/IGF-1R signaling pathway stands as a major factor in the development of photocarcinogenesis. Over a lifetime, advanced age and UVB exposure result in diminished dermal IGF-1 and increased keratinocyte mutagenesis. This results in defective DNA repair, checkpoint signaling, and appropriate keratinocyte senescence, which increases the likelihood of neoplastic transformation. NMSC remains a significant driver of increased healthcare costs and morbidity annually, necessitating the identification of effective treatments. With this new model outlined in **Figure 2**, wounding therapies hold promise as evidenced by their ability to restore appropriate IGF-1/IGF-1R signaling to levels found in younger skin. These studies are exciting as they exhibit desirable cosmetic outcomes, efficacious photorejuvation, and protection against skin carcinogenesis. As a commercially available device, microneedling holds promise as a less expensive and more widely available wounding therapy. Chemical peeling offers a beneficial alternative treatment, yet more research is needed to explicate its exact mechanism. Importantly, FLR exhibits both regenerative and long-term protective effects against pre-cancerous lesions. The concept of restoring youth and combating malignancy through wounding therapies holds potential as a major dermatological treatment modality.

The insights leading to this new paradigm of actinic neoplasia began as pioneering *in vitro* studies in which Dr. Dan Spandau discovered that keratinocyte responses to UVB were dependent upon the presence/absence of IGF-1 (99). Subsequently, Drs. Spandau and Travers devised a strategy to ascertain the importance of IGF-1R activation in the response of geriatric skin to UVB irradiation. These studies led to further collaborations with clinicians (e.g., Drs. Rohan, Tanzi, Wanner) to define the role of IGF-1 and fibroblast senescence in humans. Basic scientist Dr. Kemp has further characterized the exact mechanisms by which IGF-1R activation of the keratinocyte are protective. This new paradigm is clearly an example of a “bench-to-bedside” highly collaborative project with tremendous clinical implications.

## Author Contributions

TF wrote the first draft. CR, DS, MK, MW, ET, and JT edited and provided further input. The conceptualization of the work was from DS, JT, CR, MK, MW, ET. All authors contributed to the article and approved the submitted version.

## Funding

This work was supported by grants from the National Institutes of Health (R01 HL062996 to JT; R01 AG048946 to JT and DFS; and R01 GM130583 to MGK) and VA Merit Awards (5I01CX000809 to JT, 1I01CX002242 to MK, and 5I01CX001956 to DS).

## Author Disclaimer

The content is solely the responsibility of the authors and does not necessarily represent the official views of the National Institutes of Health nor the US Veterans Administration.

## Conflict of Interest

MW has a grant and equipment from Solta.

The remaining authors declare that the research was conducted in the absence of any commercial or financial relationships that could be construed as a potential conflict of interest.

## Publisher’s Note

All claims expressed in this article are solely those of the authors and do not necessarily represent those of their affiliated organizations, or those of the publisher, the editors and the reviewers. Any product that may be evaluated in this article, or claim that may be made by its manufacturer, is not guaranteed or endorsed by the publisher.

## Abbreviations

NMSC, Non-melanoma skin cancer; SCC, squamous cell carcinoma; BCC, basal cell carcinoma; AK, actinic keratosis; IGF-1, insulin-like growth factor-1; IGF-1R, insulin-like growth factor-1 receptor; FLR, fractionated laser resurfacing; UV, ultraviolet; NER, nucleotide excision repair; TLS, translesion synthesis.

## References

[1] Thiele JJ Podda M Packer L. Tropospheric Ozone: An Emerging Environmental Stress to Skin. Biol Chem (1997) 378(11):1299–305. doi: 10.1515/bchm.1997.378.11.1299 9426190

[2] Dyer JM Miller RA. Chronic Skin Fragility of Aging: Current Concepts in the Pathogenesis, Recognition, and Management of Dermatoporosis. J Clin Aesthet Dermatol (2018) 11(1):13–8. PMC5788262 29410724

[3] Ibuki A Kuriyama S Toyosaki Y Aiba M Hidaka M Horie Y. Aging-Like Physiological Changes in the Skin of Japanese Obese Diabetic Patients. SAGE Open Med (2018) 6:2050312118756662. doi: 10.1177/2050312118756662 29449943 PMC5808963

[4] Lephart ED. A Review of the Role of Estrogen in Dermal Aging and Facial Attractiveness in Women. J Cosmet Dermatol (2018) 17(3):282–8. doi: 10.1111/jocd.12508 29436770

[5] Society AC. Cancer Facts & Figures 2021. Atlanta: American Cancer Society (2021).

[6] Guy GP Jr. Thomas CC Thompson T Watson M Massetti GM Richardson LC. Vital Signs: Melanoma Incidence and Mortality Trends and Projections - United States, 1982-2030. MMWR Morb Mortal Wkly Rep (2015) 64(21):591–6. PMC4584771 26042651

[7] Rogers HW Weinstock MA Feldman SR Coldiron BM. Incidence Estimate of Nonmelanoma Skin Cancer (Keratinocyte Carcinomas) in the U.S. Population, 2012. JAMA Dermatol (2015) 151(10):1081–6. doi: 10.1001/jamadermatol.2015.1187 25928283

[8] Krbanjevic A Travers JB Spandau DF. How Wounding *via* Lasers Has Potential Photocarcinogenic Preventative Effects *via* Dermal Remodeling. Curr Dermatol Rep (2016) 5(3):222–7. doi: 10.1007/s13671-016-0143-8 PMC5103639 27840776

[9] Stern RS. Prevalence of a History of Skin Cancer in 2007: Results of an Incidence-Based Model. Arch Dermatol (2010) 146(3):279–82. doi: 10.1001/archdermatol.2010.4 20231498

[10] Godar DE Urbach F Gasparro FP van der Leun JC. UV Doses of Young Adults. Photochem Photobiol (2003) 77(4):453–7. doi: 10.1562/0031-8655(2003)077<0453:UDOYA>2.0.CO;2 12733658

[11] Lewis DA Travers JB Spandau DF. A New Paradigm for the Role of Aging in the Development of Skin Cancer. J Invest Dermatol (2009) 129(3):787–91. doi: 10.1038/jid.2008.293 PMC3989529 18818672

[12] Lewis DA Travers JB Somani A-K Spandau DF. The IGF-1/IGF-1R Signaling Axis in the Skin: A New Role for the Dermis in Aging-Associated Skin Cancer. Oncogene (2010) 29(10):1475–85. doi: 10.1038/onc.2009.440 PMC2837099 19966862

[13] Lewis DA Krbanjevic A Travers JB Spandau DF. “Aging-Associated Nonmelanoma Skin Cancer: A Role for the Dermis”. In: Farage M MK Maibach H, editors. Textbook of Aging Skin. Berlin, Heidelberg: Springer (2017).

[14] Tyrrell RM. The Molecular and Cellular Pathology of Solar Ultraviolet Radiation. Mol Aspects Med (1994) 15(1):1–77. 7934671

[15] Clingen PH Arlett CF Roza L Mori T Nikaido O Green MH. Induction of Cyclobutane Pyrimidine Dimers, Pyrimidine(6-4)Pyrimidone Photoproducts, and Dewar Valence Isomers by Natural Sunlight in Normal Human Mononuclear Cells. Cancer Res (1995) 55(11):2245–8. 7757971

[16] Wikonkal NM Brash DE. Ultraviolet Radiation Induced Signature Mutations in Photocarcinogenesis. J Investig Dermatol Symp Proc (1999) 4(1):6–10. doi: 10.1038/sj.jidsp.5640173 10537000

[17] Ikehata H Ono T. The Mechanisms of UV Mutagenesis. J Radiat Res (2011) 52(2):115–25. doi: 10.1269/jrr.10175 21436607

[18] Lewis DA Yi Q Travers JB Spandau DF. UVB-Induced Senescence in Human Keratinocytes Requires a Functional Insulin-Like Growth Factor-1 Receptor and P53. Mol Biol Cell (2008) 19(4):1346–53. doi: 10.1091/mbc.e07-10-1041 PMC2291419 18216278

[19] Lewis DA Travers JB Machado C Somani AK Spandau DF. Reversing the Aging Stromal Phenotype Prevents Carcinoma Initiation. Aging (Albany NY) (2011) 3(4):407–16. doi: 10.18632/aging.100318 PMC3117456 21515933

[20] Spandau DF Chen R Wargo JJ Rohan CA Southern D Zhang A. Randomized Controlled Trial of Fractionated Laser Resurfacing on Aged Skin as Prophylaxis Against Actinic Neoplasia. J Clin Invest (2021) 131(19):e150972. doi: 10.1172/JCI150972 34428179 PMC8483749

[21] Spandau DF Lewis DA Somani A-K Travers JB. Fractionated Laser Resurfacing Corrects the Inappropriate UVB Response in Geriatric Skin. J Invest Dermatol (2012) 132(6):1591–6. doi: 10.1038/jid.2012.29 PMC3352957 22377757

[22] Travers JB Kemp MG Weir NM Cates E Alkawar AM Mahajan AS. Wounding With a Microneedling Device Corrects the Inappropriate Ultraviolet B Radiation Response in Geriatric Skin. Arch Dermatol Res (2020) 312(1):1–4. doi: 10.1007/s00403-019-02001-z 31659432 PMC6989043

[23] Chen R Wargo JJ Williams A Cates E Spandau DF Knisely C. Single Ablative Fractional Resurfacing Laser Treatment For Forearm Actinic Keratoses: 6-Month Follow-Up Data From An Intrapatient Comparison Between Treated and Untreated Sites. Lasers Surg Med (2020) 52(1):84–7. doi: 10.1002/lsm.23175 PMC6982525 31736123

[24] Muzic JG Schmitt AR Wright AC Alniemi DT Zubair AS Lourido JMO. Incidence and Trends of Basal Cell Carcinoma and Cutaneous Squamous Cell Carcinoma: A Population-Based Study in Olmsted County, Minnesota, 2000 to 2010. Mayo Clin Proc (2017) 92(6):890–8. doi: 10.1016/j.mayocp.2017.02.015 PMC5535132 28522111

[25] Guy GP Jr. Machlin SR Ekwueme DU Yabroff KR. Prevalence and Costs of Skin Cancer Treatment in the U.S., 2002-2006 and 2007-2011. Am J Prev Med (2015) 48(2):183–7. doi: 10.1016/j.amepre.2014.08.036 PMC4603424 25442229

[26] Samarasinghe V Madan V. Nonmelanoma Skin Cancer. J Cutan Aesthet Surg (2012) 5(1):3–10. doi: 10.4103/0974-2077.94323 22557848 PMC3339125

[27] Gordon R. Skin Cancer: An Overview of Epidemiology and Risk Factors. Semin Oncol Nurs (2013) 29(3):160–9. doi: 10.1016/j.soncn.2013.06.002 23958214

[28] Kraemer KH. Sunlight and Skin Cancer: Another Link Revealed. Proc Natl Acad Sci USA (1997) 94(1):11–4. doi: 10.1073/pnas.94.1.11 PMC33651 8990152

[29] Barysch MJ Hofbauer GF Dummer R. Vitamin D, Ultraviolet Exposure, and Skin Cancer in the Elderly. Gerontology (2010) 56(4):410–3. doi: 10.1159/000315119 20502035

[30] Brash DE NP. “Carcinogenesis: Ultraviolet Radiation”. In: Wolff, editor. Fitzpatrick’s Dermatology in General Medicine (2003). New York: McGraw-Hill Professional.

[31] Zak-Prelich M Narbutt J Sysa-Jedrzejowska A. Environmental Risk Factors Predisposing to the Development of Basal Cell Carcinoma. Dermatol Surg (2004) 30(2 Pt 2):248–52. doi: 10.1111/j.1524-4725.2004.30089.x 14871217

[32] Rosso S Zanetti R Martinez C Tormo MJ Schruab S Sancho-Garnier H. The Multicentre South European Study ‘Helios’. II: Different Sun Exposure Patterns in the Aetiology of Basal Cell and Squamous Cell Carcinomas of the Skin. Br J Cancer (1996) 73(11):1447–54. doi: 10.1038/bjc.1996.275 PMC2074492 8645596

[33] Kricker A Armstrong BK English DR Heenan PJ. Does Intermittent Sun Exposure Cause Basal Cell Carcinoma? A Case-Control Study in Western Australia. Int J Cancer (1995) 60(4):489–94. doi: 10.1002/ijc.2910600411 7829262

[34] Roewert-Huber J Lange-Asschenfeldt B Stockfleth E Kerl H. Epidemiology and Aetiology of Basal Cell Carcinoma. Br J Dermatol (2007) 157 Suppl 2:47–51. doi: 10.1111/j.1365-2133.2007.08273.x 18067632

[35] Whiteman DC Whiteman CA Green AC. Childhood Sun Exposure as a Risk Factor for Melanoma: A Systematic Review of Epidemiologic Studies. Cancer Causes Control (2001) 12(1):69–82. doi: 10.1023/A:1008980919928 11227927

[36] Kricker A Armstrong BK English DR. Sun Exposure and Non-Melanocytic Skin Cancer. Cancer Causes Control (1994) 5(4):367–92. doi: 10.1007/BF01804988 8080949

[37] Westerdahl J Olsson H Ingvar C. At What Age do Sunburn Episodes Play a Crucial Role for the Development of Malignant Melanoma. Eur J Cancer (1994) 30A(11):1647–54. doi: 10.1016/0959-8049(94)00337-5 7833138

[38] Narayanan DL Saladi RN Fox JL. Ultraviolet Radiation and Skin Cancer. Int J Dermatol (2010) 49(9):978–86. doi: 10.1111/j.1365-4632.2010.04474.x 20883261

[39] Thompson SC Jolley D Marks R. Reduction of Solar Keratoses by Regular Sunscreen Use. N Engl J Med (1993) 329(16):1147–51. doi: 10.1056/NEJM199310143291602 8377777

[40] Naylor MF Boyd A Smith DW Cameron GS Hubbard D Neldner KH. High Sun Protection Factor Sunscreens in the Suppression of Actinic Neoplasia. Arch Dermatol (1995) 131(2):170–5. doi: 10.1001/archderm.1995.01690140054008 7857113

[41] Ulrich C Jurgensen JS Degen A Hackethal M Ulrich M Patel MJ. Prevention of Non-Melanoma Skin Cancer in Organ Transplant Patients by Regular Use of a Sunscreen: A 24 Months, Prospective, Case-Control Study. Br J Dermatol (2009) 161 Suppl 3:78–84. doi: 10.1111/j.1365-2133.2009.09453.x 19775361

[42] Freitas AA de Magalhaes JP. A Review and Appraisal of the DNA Damage Theory of Ageing. Mutat Res (2011) 728(1-2):12–22. doi: 10.1016/j.mrrev.2011.05.001 21600302

[43] Hoeijmakers JH. DNA Damage, Aging, and Cancer. N Engl J Med (2009) 361(15):1475–85. doi: 10.1056/NEJMra0804615 19812404

[44] Gruber F Kremslehner C Eckhart L Tschachler E. Cell Aging and Cellular Senescence in Skin Aging - Recent Advances in Fibroblast and Keratinocyte Biology. Exp Gerontol (2020) 130:110780. doi: 10.1016/j.exger.2019.110780 31794850

[45] Eckhart L Zeeuwen P. The Skin Barrier: Epidermis vs Environment. Exp Dermatol (2018) 27(8):805–6. doi: 10.1111/exd.13731 29989217

[46] Tigges J Krutmann J Fritsche E Haendeler J Schaal H Fischer JW. The Hallmarks of Fibroblast Ageing. Mech Ageing Dev (2014) 138:26–44. doi: 10.1016/j.mad.2014.03.004 24686308

[47] Goubran HA Kotb RR Stakiw J Emara ME Burnouf T. Regulation of Tumor Growth and Metastasis: The Role of Tumor Microenvironment. Cancer Growth Metastasis (2014) 7:9–18. doi: 10.4137/CGM.S11285 24926201 PMC4051818

[48] Moriwaki S Takahashi Y. Photoaging and DNA Repair. J Dermatol Sci (2008) 50(3):169–76. doi: 10.1016/j.jdermsci.2007.08.011 17920816

[49] Coppe JP Desprez PY Krtolica A Campisi J. The Senescence-Associated Secretory Phenotype: The Dark Side of Tumor Suppression. Annu Rev Pathol (2010) 5:99–118. doi: 10.1146/annurev-pathol-121808-102144 20078217 PMC4166495

[50] Ghosh K Capell BC. The Senescence-Associated Secretory Phenotype: Critical Effector in Skin Cancer and Aging. J Invest Dermatol (2016) 136(11):2133–9. doi: 10.1016/j.jid.2016.06.621 PMC5526201 27543988

[51] Waldera Lupa DM Kalfalah F Safferling K Boukamp P Poschmann G Volpi E. Characterization of Skin Aging-Associated Secreted Proteins (SAASP) Produced by Dermal Fibroblasts Isolated From Intrinsically Aged Human Skin. J Invest Dermatol (2015) 135(8):1954–68. doi: 10.1038/jid.2015.120 25815425

[52] Loesch MM Collier AE Southern DH Ward RE Tholpady SS Lewis DA. Insulin-Like Growth Factor-1 Receptor Regulates Repair of Ultraviolet B-Induced DNA Damage in Human Keratinocytes *In Vivo* . Mol Oncol (2016) 10(8):1245–54. doi: 10.1016/j.molonc.2016.06.002 PMC5026895 27373487

[53] Melnikova VO Ananthaswamy HN. Cellular and Molecular Events Leading to the Development of Skin Cancer. Mutat Res (2005) 571(1-2):91–106. doi: 10.1016/j.mrfmmm.2004.11.015 15748641

[54] Madan V Lear JT Szeimies RM. Non-Melanoma Skin Cancer. Lancet (2010) 375(9715):673–85. doi: 10.1016/S0140-6736(09)61196-X 20171403

[55] Drouin R Therrien JP. UVB-Induced Cyclobutane Pyrimidine Dimer Frequency Correlates With Skin Cancer Mutational Hotspots in P53. Photochem Photobiol (1997) 66(5):719–26. doi: 10.1111/j.1751-1097.1997.tb03213.x 9383997

[56] Ichihashi M Ueda M Budiyanto A Bito T Oka M Fukunga M. UV-Induced Skin Damage. Toxicology (2003) 189(1-2):21–39. doi: 10.1016/S0300-483X(03)00150-1 12821280

[57] Nishigori C. Cellular Aspects of Photocarcinogenesis. Photochem Photobiol Sci (2006) 5(2):208–14. doi: 10.1039/B507471A 16465307

[58] Krtolica A Campisi J. Cancer and Aging: A Model for the Cancer Promoting Effects of the Aging Stroma. Int J Biochem Cell Biol (2002) 34(11):1401–14. doi: 10.1016/S1357-2725(02)00053-5 12200035

[59] Mathon NF Lloyd AC. Cell Senescence and Cancer. Nat Rev Cancer (2001) 1(3):203–13. doi: 10.1038/35106045 11902575

[60] Campisi J. Cancer and Ageing: Rival Demons? Nat Rev Cancer (2003) 3(5):339–49. doi: 10.1038/nrc1073 12724732

[61] Ramos J Villa J Ruiz A Armstrong R Matta J. UV Dose Determines Key Characteristics of Nonmelanoma Skin Cancer. Cancer Epidemiol Biomarkers Prev (2004) 13(12):2006–11. 15598755

[62] Brash DE. Roles of the Transcription Factor P53 in Keratinocyte Carcinomas. Br J Dermatol (2006) 154 Suppl 1:8–10. doi: 10.1111/j.1365-2133.2006.07230.x 16712710

[63] Kemp MG Spandau DF Travers JB. Impact of Age and Insulin-Like Growth Factor-1 on DNA Damage Responses in UV-Irradiated Human Skin. Molecules (2017) 22(3):356–76. doi: 10.3390/molecules22030356 PMC5432641 28245638

[64] Tavakkol A Elder JT Griffiths CE Cooper KD Talwar H Fisher GJ. Expression of Growth Hormone Receptor, Insulin-Like Growth Factor 1 (IGF-1) and IGF-1 Receptor mRNA and Proteins in Human Skin. J Invest Dermatol (1992) 99(3):343–9. doi: 10.1111/1523-1747.ep12616668 1324963

[65] Kemp MG Spandau DF Simman R Travers JB. Insulin-Like Growth Factor 1 Receptor Signaling Is Required for Optimal ATR-CHK1 Kinase Signaling in Ultraviolet B (UVB)-Irradiated Human Keratinocytes. J Biol Chem (2017) 292(4):1231–9. doi: 10.1074/jbc.M116.765883 PMC5270469 27979966

[66] Hutcherson RJ Gabbard RD Castellanos AJ Travers JB Kemp MG. Age and Insulin-Like Growth Factor-1 Impact PCNA Monoubiquitination in UVB-Irradiated Human Skin. J Biol Chem (2021) 296:100570. doi: 10.1016/j.jbc.2021.100570 33753168 PMC8065225

[67] Fernandez TL Van Lonkhuyzen D Dawson R Kimlin M Upton Z. Insulin-Like Growth Factor-I and UVB Photoprotection in Human Keratinocytes. Exp Dermatol (2015) 24(3):235–8. doi: 10.1111/exd.12637 25607472

[68] Alkawar AMM Castellanos AJ Carpenter MA Hutcherson RJ Madkhali MAO Johnson RM. Insulin-Like Growth Factor-1 Impacts P53 Target Gene Induction in UVB-Irradiated Keratinocytes and Human Skin. Photochem Photobiol (2020) 96(6):1332–41. doi: 10.1111/php.13279 PMC8674807 32416609

[69] Lazareth V. Management of Non-Melanoma Skin Cancer. Semin Oncol Nurs (2013) 29(3):182–94. doi: 10.1016/j.soncn.2013.06.004 23958216

[70] Amaral T Garbe C. Non-Melanoma Skin Cancer: New and Future Synthetic Drug Treatments. Expert Opin Pharmacother (2017) 18(7):689–99. doi: 10.1080/14656566.2017.1316372 28414587

[71] Griffin LL Ali FR Lear JT. Non-Melanoma Skin Cancer. Clin Med (Lond) (2016) 16(1):62–5. doi: 10.7861/clinmedicine.16-1-62 PMC4954336 26833519

[72] Ceovic R Petkovic M Mokos ZB Kostovic K. Nonsurgical Treatment of Nonmelanoma Skin Cancer in the Mature Patient. Clin Dermatol (2018) 36(2):177–87. doi: 10.1016/j.clindermatol.2017.10.009 29566922

[73] Malvehy J. A New Vision of Actinic Keratosis Beyond Visible Clinical Lesions. J Eur Acad Dermatol Venereol (2015) 29 Suppl 1:3–8. doi: 10.1111/jdv.12833 25470718

[74] Schmitt AR Bordeaux JS. Solar Keratoses: Photodynamic Therapy, Cryotherapy, 5-Fluorouracil, Imiquimod, Diclofenac, or What? Facts and Controversies. Clin Dermatol (2013) 31(6):712–7. doi: 10.1016/j.clindermatol.2013.05.007 24160275

[75] Gupta AK Paquet M Villanueva E Brintnell WL. Interventions for Actinic Keratoses. Cochrane Database Syst Rev (2012) 12:CD004415. doi: 10.1002/14651858.CD004415.pub2 23235610 PMC6599879

[76] Rosenberg AR Tabacchi M Ngo KH Wallendorf M Rosman IS Cornelius LA. Skin Cancer Precursor Immunotherapy for Squamous Cell Carcinoma Prevention. JCI Insight (2019) 4(6):e125476. doi: 10.1172/jci.insight.125476 PMC6483001 30895944

[77] Travers JB Spandau DF Lewis DA Machado C Kingsley M Mousdicas N. Fibroblast Senescence and Squamous Cell Carcinoma: How Wounding Therapies Could be Protective. Dermatol Surg (2013) 39(7):967–73. doi: 10.1111/dsu.12138 PMC4112094 23437969

[78] Loesch MM Somani AK Kingsley MM Travers JB Spandau DF. Skin Resurfacing Procedures: New and Emerging Options. Clin Cosmet Investig Dermatol (2014) 7:231–41. doi: 10.2147/CCID.S50367 PMC4155739 25210469

[79] Alkhawam L Alam M. Dermabrasion and Microdermabrasion. Facial Plast Surg (2009) 25(5):301–10. doi: 10.1055/s-0029-1243078 20024871

[80] Gold MH. Dermabrasion in Dermatology. Am J Clin Dermatol (2003) 4(7):467–71. doi: 10.2165/00128071-200304070-00003 12814336

[81] Roenigk HH. Dermabrasion: State of the Art 2002. J Cosmet Dermatol (2002) 1(2):72–87. doi: 10.1046/j.1473-2165.2002.00041.x 17147523

[82] Hamilton MM Kao R. Recognizing and Managing Complications in Laser Resurfacing, Chemical Peels, and Dermabrasion. Facial Plast Surg Clin North Am (2020) 28(4):493–501. doi: 10.1016/j.fsc.2020.06.008 33010868

[83] Carniol PJ Hamilton MM Carniol ET. Current Status of Fractional Laser Resurfacing. JAMA Facial Plast Surg (2015) 17(5):360–6. doi: 10.1001/jamafacial.2015.0693 26133312

[84] Aslam A Alster TS. Evolution of Laser Skin Resurfacing: From Scanning to Fractional Technology. Dermatol Surg (2014) 40(11):1163–72. doi: 10.1097/01.DSS.0000452648.22012.a0 25285818

[85] Alexiades-Armenakas MR Dover JS Arndt KA. The Spectrum of Laser Skin Resurfacing: Nonablative, Fractional, and Ablative Laser Resurfacing. J Am Acad Dermatol (2008) 58(5):719–37; quiz 738-40. doi: 10.1016/j.jaad.2008.01.003 18423256

[86] Alster TS Graham PM. Microneedling: A Review and Practical Guide. Dermatol Surg (2018) 44(3):397–404. doi: 10.1097/DSS.0000000000001248 28796657

[87] Alessa D Bloom JD. Microneedling Options for Skin Rejuvenation, Including Non-Temperature-Controlled Fractional Microneedle Radiofrequency Treatments. Facial Plast Surg Clin North Am (2020) 28(1):1–7. doi: 10.1016/j.fsc.2019.09.001 31779933

[88] Mdanda S Ubanako P Kondiah PPD Kumar P Choonara YE. Recent Advances in Microneedle Platforms for Transdermal Drug Delivery Technologies. Polymers (Basel) (2021) 13(15):2405–29. doi: 10.3390/polym13152405 PMC8348894 34372008

[89] Lee KC Wambier CG Soon SL Sterling JB Landau M Rullan P. Basic Chemical Peeling: Superficial and Medium-Depth Peels. J Am Acad Dermatol (2019) 81(2):313–24. doi: 10.1016/j.jaad.2018.10.079 30550830

[90] Sidiropoulou P Gregoriou S Rigopoulos D Kontochristopoulos G. Chemical Peels in Skin Cancer: A Review. J Clin Aesthet Dermatol (2020) 13(2):53–7. PMC7158909 32308785

[91] Starkman SJ Mangat DS. Chemical Peels: Deep, Medium, and Light. Facial Plast Surg (2019) 35(3):239–47. doi: 10.1055/s-0039-1688944 31189196

[92] Soleymani T Lanoue J Rahman Z. A Practical Approach to Chemical Peels: A Review of Fundamentals and Step-By-Step Algorithmic Protocol for Treatment. J Clin Aesthet Dermatol (2018) 11(8):21–8. PMC6122508 30214663

[93] Ahn KS Park KS Jung KM Jung HK Lee SH Chung SY. Inhibitory Effect of Glycolic Acid on Ultraviolet B-Induced C-Fos Expression, AP-1 Activation and P53-P21 Response in a Human Keratinocyte Cell Line. Cancer Lett (2002) 186(2):125–35. doi: 10.1016/S0304-3835(02)00283-5 12213282

[94] Kaminaka C Yamamoto Y Yonei N Kishioka A Kondo T Furukawa F. Phenol Peels as a Novel Therapeutic Approach for Actinic Keratosis and Bowen Disease: Prospective Pilot Trial With Assessment of Clinical, Histologic, and Immunohistochemical Correlations. J Am Acad Dermatol (2009) 60(4):615–25. doi: 10.1016/j.jaad.2008.11.907 19293009

[95] Lawrence N Cox SE Cockerell CJ Freeman RG Cruz PD Jr. A Comparison of the Efficacy and Safety of Jessner’s Solution and 35% Trichloroacetic Acid vs 5% Fluorouracil in the Treatment of Widespread Facial Actinic Keratoses. Arch Dermatol (1995) 131(2):176–81. doi: 10.1001/archderm.1995.01690140060009 7857114

[96] Hantash BM Stewart DB Cooper ZA Rehmus WE Koch RJ Swetter SM. Facial Resurfacing for Nonmelanoma Skin Cancer Prophylaxis. Arch Dermatol (2006) 142(8):976–82. doi: 10.1001/archderm.142.8.976 16924046

[97] Di Nuzzo S Cortelazzi C Boccaletti V Zucchi A Conti ML Montanari P. Comparative Study of Trichloroacetic Acid vs. Photodynamic Therapy With Topical 5-Aminolevulinic Acid for Actinic Keratosis of the Scalp. Photodermatol Photoimmunol Photomed (2015) 31(5):233–8. doi: 10.1111/phpp.12164 25660106

[98] Holzer G Pinkowicz A Radakovic S Schmidt JB Tanew A. Randomized Controlled Trial Comparing 35% Trichloroacetic Acid Peel and 5-Aminolaevulinic Acid Photodynamic Therapy for Treating Multiple Actinic Keratosis. Br J Dermatol (2017) 176(5):1155–61. doi: 10.1111/bjd.15272 28012181

[99] Kuhn C Hurwitz SA Kumar MG Spandau DF. Activation of the Insulin-Like Growth Factor-1 Receptor Promotes the Survival of Human Keratinocytes Following Ultraviolet B Radiation. Int J Cancer (1999) 80(3):431–8. doi: 10.1002/(SICI)1097-0215(19990129)80:3<431::AID-IJC16>3.0.CO;2-5 9935186

